# Endoscopic Management of Peri-Pancreatic Collections

**DOI:** 10.1155/2012/906381

**Published:** 2012-02-09

**Authors:** David Keith Swartz, Jorge Obando

**Affiliations:** Division of Gastroenterology, Department of Medicine, Duke University Medical Center, Durham, NC, USA

## Abstract

Endotherapy of peripancreatic fluid collections is an increasing utilized procedure in interventional endoscopy. The aim of this paper is to provide a general overview of the topic, highlighting the indications, technique, and important management issues relating to endoscopic management of the various forms of peri-pancreatic fluid collections.

## 1. Introduction

Acute pancreatitis is a common clinical problem, with an incidence of 210,000 per year in the USA [1], and is most commonly a consequence of biliary tract stones and alcohol. There is a wide spectrum of disease severity ranging from mild self-limiting disease to severe acute pancreatitis (SAP), an entity characterized by the presence of systemic and local complications. Local complications include peri-pancreatic collections, which can result in significant morbidity and mortality. Chronic pancreatitis is also a common problem causing peri-pancreatic collections, in particular pseudocysts. For many years, surgery was the major modality used to treat these entities. Endoscopic trans-gastric treatment of pseudocysts evolved in the late 1970's [2]. Percutaneous radiologically assisted procedures were also employed [3, 4] and, with the advent and development of cross-sectional imaging, were used more frequently. Endoscopic trans-gastric or trans-duodenal drainage established a role for endotherapy in the management of these problems following the publication of success in a large series [5]. The use of endosonography in the management of peri-pancreatic collections is a more recent development, but one that has rapidly evolved since its first reported use in drainage of pancreatic pseudocysts in 1992 [6]. With EUS assistance, indications for the use of endoscopic therapy were extended to include pancreatic necrosectomy in 1996 [7].

## 2. Classification of Peri-Pancreatic Collections

A consensus conference in 1992 established the currently accepted definitions of the local complications of pancreatitis in what is known as the Atlanta Classification [8]. Five major morphological entities were defined—pancreatic necrosis, acute fluid collections, pancreatic abscess, acute pseudocyst, and chronic pseudocyst. Pancreatic necrosis features diffuse or focal areas of non-viable pancreatic parenchyma, usually with associated peri-pancreatic fat necrosis. Acute fluid collections are peri-pancreatic collections of fluid, located in or near the pancreas, lacking a wall of surrounding granulation or fibrous tissue, arising more than 4 weeks after an episode of acute pancreatitis. Pancreatic abscess is a circumscribed intra-abdominal area of pus, usually in proximity to the pancreas, containing little or no pancreatic necrosis. Acute pseudocyst is a collection of pancreatic juice enclosed by a wall of fibrous or granulation tissue. An additional entity of organised pancreatic necrosis (OPN), or walled-off pancreatic necrosis (WOPN), is frequently referred to in the literature, although it is not a specific entity in the Atlanta Classification. This refers to a collection with good separation of devitalised (necrotic) tissue within a fluid-filled cavity, and an associated fibrous wall lined by granulation tissue [9], as distinct from pancreatic necrosis that is not well defined and lacks a wall (Table 1).

These morphological manifestations result from the underlying inflammation associated with acute pancreatitis. In the case of acute fluid collections, serous or exudative reactions develop in response to inflammation early in the course of disease and are not in communication with the pancreatic ductal system [10]. Acute pseudocysts are a consequence of inflammation-induced pancreatic ductal disruption, with extravasation of pancreatic juice inducing the formation of a fibrous wall in an attempt to contain the fluid [8].

## 3. Clinical Manifestations/Indications for Intervention

### 3.1. Mass Effect

All peri-pancreatic collections can cause symptoms by virtue of their mass effect and potential to enlarge. Pain and abdominal fullness are common symptoms. Compression of adjacent organs like the stomach, duodenum, and bile duct can result in early satiety, vomiting, weight loss, and jaundice [11]. Symptoms resulting from the mass effect of fluid collections, in particular pseudocysts, are frequently the precipitant for endoscopic treatment.

### 3.2. Infection

All peri-pancreatic collections have the potential to become infected, spontaneously through translocation of normal gut flora, and via iatrogenic means in the setting of instrumentation of a previously sterile collection of tissue or fluid. As expected, enteric gram-negative organisms, in particular *Enterococcus*, *Pseudomonas*, and *Klebsiella*, tend to be the most commonly implicated [12], although *Candida* species are not uncommonly associated [13].

Infected pseudocysts, pancreatic abscesses, and infected necrosis are all potentially amenable to endoscopic management with the aim of removing the infected focus.

### 3.3. Hemorrhage/Perforation

Hemorrhage is an uncommon but serious complication of pancreatic fluid collections. Compression of an adjacent vessel and subsequent erosion can result in pseudoaneurysm formation, with potential for rupture and hemorrhage. The most common site for pseudoaneurysm formation is the splenic artery. Rupture of an aneurysm into a pseudocyst or WOPN can result in hemosuccus pancreaticus [14]. Perforations of pseudocysts into the peritoneum and bowel are well described although uncommon potential complications [15, 16].

### 3.4. Size

Non-randomized data from published series have identified pseudocyst size as a significant predictor of need for intervention, with pseudocysts of greater than 6 cm in size requiring intervention in more than 2/3 of cases [17]. On the basis of this, some advocate treatment of cysts greater than 6 cm in size, even in the absence of other clinical symptoms. However, series with long-term follow-up suggest that asymptomatic cysts can usually be managed conservatively, regardless of size.

## 4. Therapeutic Modalities

### 4.1. Surgery

Surgical management has long been considered the gold-standard for management of peri-pancreatic collections, and was the main treatment modality for many years, prior to the availability of less invasive options. In general, surgical management provides direct access to the peri-pancreatic collection with multiple means of drainage, at the cost of being more invasive and universally requiring general anesthesia. For surgical management of pseudocysts, open external drainage is now generally not deemed appropriate, and internal drainage via formation of a cystgastrostomy or cystenterostomy is the preferred option [18]. Success rates with resolution of cysts using surgical methods is high, but with relatively high complication rates in the range of 24–40% [19–22] and mortality of 5.8% [19]. Laparoscopic approaches have been used more recently, although there is little data comparing outcomes with the open approach. These procedures are associated with around a 10% conversion rate, but a much lower complication and mortality rate of around 12% and 1% respectively [23–27].

### 4.2. Percutaneous Radiological Drainage

Percutaneous drainage of pseudocysts and abscesses is most commonly performed under CT guidance, with either external (direct puncture of the cyst through the anterior abdominal wall) or internal drainage (direct puncture with placement of double-pigtail stents to form a cystgastrostomy under fluoroscopic or endoscopic guidance).

External drainage has a significant failure rate, particularly in the setting of abnormal pancreatic ductal anatomy, and rates of cutaneous fistula formation have been reported to be as high as 50% in certain settings [28].

With the internal technique, success in catheter placement is usually achievable more than 90% of the time, with an immediate complication rate of around 6% and a mortality rate of 1%. Secondary infection with abscess formation is not uncommon, occurring in around 11% [29]. Complete resolution of the pseudocyst with this method has been reported as 88% or more in several small series [30, 31].

### 4.3. Endoscopic Therapy

#### 4.3.1. Pseudocysts

Reported success rates for endoscopic treatment of pseudocysts are very high, ranging from 91 to 100% in the published literature [32–34]. Better technical success rates are achieved with EUS-guided procedures.

#### 4.3.2. Abscess

By the Atlanta Classification, a pancreatic abscess contains no solid necrotic material. Whilst this situation results in high clinical success rates with endoscopic management, such a situation is actually quite uncommon, as the majority of infected peri-pancreatic fluid collections tend to be associated with some degree of necrosis [10]. Because of this, data on outcomes for abscess drainage is limited. What does appear apparent is that, compared with pseudocyst drainage, endoscopic management has a lower success rate and a higher rate of complications. Older retrospective data suggested no difference in outcome with pseudocyst and abscess drainage [35]. In a recent prospective study, the success rate for abscess drainage was 80%, with a complication rate of 30%, mainly in the form of perforation [36]. This data supports a more cautious approach to abscesses compared with pseudocysts.

#### 4.3.3. Pancreatic Necrosis

The technique of endoscopic necrosectomy for pancreatic necrosis has evolved over the last 10 year. With increased experience, outcomes have significantly improved, to the point that this technique has superseded other forms of intervention as the first-line management where technically feasible. Reported rates of successful resolution of walled-off pancreatic necrosis exceed 75%, with a relatively low mortality rate. In up to 20% or more of cases, walled-off necrosis persists despite endoscopic management, and surgical or radiological intervention may still be required [12, 13]. The most recent data comes from a large multicenter retrospective series of 104 patients examining endoscopic necrosectomy for symptomatic necrosis [37]. This study reported successful resolution of pancreatic necrosis in 91%, with a complication rate of 14%.

Certain disease and patient-related factors have been shown to impart a higher failure rate in endoscopic necrosectomy and should influence decisions regarding the management modality used. Extension of the collection into the paracolic gutters, a collection size greater than 15 cm, and patients with comorbid diabetes mellitus have all been associated with lower success rates with endoscopic therapy [12].

### 4.4. Comparison of Therapeutic Modalities

Few studies have set out to directly compare the therapeutic modalities available for management of peri-pancreatic fluid collections. Most of those that have were small in size, and there are no randomized studies comparing the various techniques.

A large population-based analysis comparing surgical with percutaneous management of pancreatic pseudocysts [20] demonstrated significant differences in favor of surgical management in relation to complications, length of stay (15 days versus 21 days), and inpatient mortality (2.8% versus 5.9%).

Generally, the choice of therapeutic modality employed depends on the characteristics of the collection, patient factors, local expertise, and physician preference.

## 5. Assessment Prior to Endoscopic Therapy

An adequate assessment of the nature and anatomical relations of peri-pancreatic fluid collections is necessary prior to endoscopic therapy, in order to maximize success and minimize complication rates.

### 5.1. Cross-Sectional Imaging

Imaging with CT or MRI provides important information about the nature of peri-pancreatic fluid collections in a non-invasive fashion. Contrast-enhanced CT scanning is the most commonly employed technique and is able to provide information on size of the collection, presence and thickness of a wall, presence of internal debris, and contrast-enhancement characteristics that suggest tissue necrosis. Contrast-enhanced, multidetector row CT scan is the best imaging modality to exclude alternative diagnoses, assess severity, and identify complications [38]. Pancreatic MRI is an alternative imaging modality, with evidence to suggest a greater ability to detect solid components within a fluid collection and therefore better distinguish pseudocysts from walled-off necrosis [39]. This advantage means that MRI assessment should be strongly considered as part of the workup of pancreatic fluid collections prior to endotherapy. The presence of a mature wall is a prerequisite for endoscopic intervention, and so pre-intervention imaging guides decision-making on the timing and appropriateness of potential management strategies. MRCP can provide important details on the relationship of peri-pancreatic fluid collections to the pancreatic duct and identify pathology associated with a disconnected pancreatic tail. If a collection can be demonstrated to communicate with the PD, endoscopic management may need to address the pancreatic fistula, in addition to the cyst itself (see Section 6.3). 

### 5.2. Use of Endosonography

Although utilization of EUS to assess peri-pancreatic fluid collections is not essential, it has been demonstrated to provide multiple advantages.

The use of EUS is indicated to visualize and avoid vessels or varices that may exist along the path of the needle or fistula to be created. It is also indicated when a cyst does not have a bulge into the GI lumen. It has traditionally been considered that 1 cm is the maximum recommended distance between the GI lumen and the cyst cavity when considering endoscopic therapy.

The addition of an EUS assessment of peri-pancreatic fluid collections prior to endoscopic intervention can result in a change in management in up to 1/3 of patients. Changes can result from alternative diagnoses other than peri-pancreatic fluids collections, and identification of anatomical and vascular factors preventing endoscopic management [40]. This is particularly important in patients with portal hypertension, where vessels are more likely to be interposed between the GI tract and the peri-pancreatic collection [41]. The presence of significant solid debris within a collection can often be seen on EUS, even in settings where radiological imaging had failed to identify such elements. Such a finding suggesting pancreatic necrosis may well change a decision to intervene in what was previously thought to be a pseudocyst, due the risk of converting sterile necrosis into infected necrosis.

For these reasons and others, studies have consistently demonstrated an increased success rate with utilization of EUS. In a randomized trial, EUS use resulted in as much as a 66% increase in success [42]. The largest randomized trial comparing the techniques demonstrated a 91% success rate with employment of EUS, compared with 72% when not used. Most failures without EUS use were due to the non-bulging nature of the collection. When EUS was used in these cases, drainage was successful [43].

## 6. Technique

### 6.1. Pseudocyst Drainage

Pseudocyst drainage is usually fairly straightforward technically when done on a cyst with no solid component. The aim is to create a permanent fistula between the cyst and the adjacent GI tract, typically the stomach or the duodenum. The location of the fistula should not matter since the fluid will empty into the GI tract by pressure effect. We try to avoid the very high stomach as stents may interfere with the GE junction, although many times this is an area that is very well visualized with the endosonoscope and appealing, given that the endoscope is straight and short.

The technique involves advancing a wire into the cyst. The cyst cavity can be pierced either with a 19-gauge EUS needle under EUS guidance or with a needle-knife if not using EUS. A wire can then be advanced under fluoroscopy into the cyst, and typically it is seen coiling inside the cyst. We feel that injection of contrast into the cyst is not always necessary. We prefer the Boston Scientific 450 cm, 0.035′ Superstiff Jagwire, which may counteract the early coiling of the double-pigtail stent as it is being deployed later in the procedure. If using the EUS needle, it is important not to pull the wire back into the needle since there is concern for shearing a piece of the wire by the sharp needle tip.

The next step after the wire is in place is to dilate the tract. We prefer to see whether a dilation balloon will be able to pierce the gastric wall into the cyst and favor the Hurricane 8–10 mm biliary dilation balloon, given its flexible tip, and stiff shaft due to the presence of a stylet. If we are unsuccessful, we then use either the needle-knife with 1-2 mm of the needle out, with ERBE sphincterotomy settings, or a cystotome (Cystotome, Wilson Cook). The tract is pierced with the thin sheath and immediately dilated with the thicker sheath, using 80–100 watts of pure-cut current.

The tract is then balloon-dilated under fluoroscopy to 10 mm for 1 minute. Typically after deflating the balloon, a large amount of turbid, yellowish fluid emanates from the tract.

In our practice, a minimum of three double-pigtail stents are then placed if possible. It is typically difficult to advance a 10 Fr stent through the endosonoscope lumen, especially if there are 2 wires in the cyst. In these cases, we either place 7 Fr stents or switch the endoscope for a therapeutic, larger-channel endoscope.

Selection of stent size depends on the size of the pseudocyst. For smaller cysts (10 cm in diameter), we prefer 10 Fr, 4 cm stents. For larger cysts, we prefer placing 10 Fr, 7 cm stents, although there is no data to guide this practice. Placing a 10 Fr double-pigtail stent can be challenging given the tendency of the stent to recover its shape as soon as it comes out of the endoscope channel, hence the use of the Superstiff Jagwire from initial cyst puncture. Care should be taken to ensure the stent is deployed correctly and does not migrate into the pseudocyst lumen.

Follow-up at our institution is typically with a CT scan at 4 weeks and a clinic visit for review, with an endoscopic stent removal procedure shortly thereafter, assuming the cyst has resolved. There has been a recent study that supports keeping stents in-situ for a longer period of time, with superior results obtained with stent durations of up to 2 years [44]. The pancreatic ductal anatomy also influences stent duration. If a disconnected tail is seen, it is likely that the disconnected pancreas will continue draining into the cyst cavity. To promote a more mature fistula and prevent early closure, we tend to leave the stents for a longer time, about three months, in these cases.

### 6.2. Necrosectomy

In the first session of necrosectomy, our technique is similar to the one described for pseudocyst management. The goal at this point is to drain the liquid component of the cyst. Stents are placed as previously described. The second session typically takes place a few days later. These procedures can be time-consuming, so they are performed under general anesthesia.

At subsequent sessions, the stents are removed and a wire is placed in the cyst cavity under fluoroscopy. The tract is dilated to 16–18 mm, and a therapeutic endoscope is advanced into the cavity guided by the wire. Care is needed not to over-insufflate the cyst cavity with air. The instruments selected to remove non-viable material will depend on whether the necrotic debris is semi-solid or solid. For liquefied or semi-solid debris, irrigation via the jet port is usually helpful, although suctioning may only clog the channel if there are large particles. In order to gain the greatest efficiency of solid tissue removal, instruments we use (in order of preference) are nets, (Nakao-Spider, ConMed; Roth, US Endoscopy), baskets (Twister, Boston Scientific), snares, or biopsy forceps to break large blocks of necrosis. This choice of instruments is subjective and depends entirely on physician preference and the nature of the material to be removed. The scope is advanced into the cavity as many times as needed to remove as much debris as possible. The stents are replaced at the end of the procedure.

The procedure is typically repeated 3-4 times, at weekly intervals, in order to obtain complete clearance of necrotic tissue. The patient is typically covered with antibiotics. Although the number of sessions required is highly variable and dependent on the nature of the cavity and extent of necrosis, studies examining the issue have found the median number of session required to be 3, with up to 12 sessions being required in some cases [12].

### 6.3. Adjunctive Therapy

#### 6.3.1. Management of the Pancreatic Duct

The etiologies of pancreatic fluid collections, particularly those that occur in the setting of acute pancreatitis, are related to disruption to the pancreatic ductal system. As a sequelae of this, pancreatic fistulas are commonly seen. Any direct communication of the pancreatic ductal system with the fluid collection means that pancreatic glandular secretions have a passage through which to enter and propagate the collection. If endoscopic drainage of a collection is performed, pancreatic fistulae are a cause of potential recurrence. As such, integrity of the pancreatic duct is a prerequisite for successful endoscopic therapy in the long term. For this reason, the importance of appropriate assessment of the anatomical relationship of the pancreatic duct to the peri-pancreatic collection prior to endoscopic treatment with MRCP or ERCP cannot be understated. Should a pancreatic fistula be identified, consideration should be given to performing trans-papillary drainage. This procedure involves passage of a hydrophilic guidewire through the pancreatic duct and either into the collection through the fistula or upstream of the fistulous tract in the pancreatic duct. A pancreatic sphincterotomy and placement of a pancreatic stent are then performed. Such intervention has been shown to improve treatment outcomes in patients undergoing endoscopic transmural drainage of a pancreatic fluid collection [45]. The duration of pancreatic stent placement depends upon the time taken for resolution of the collection and the presence of duct strictures or stones, which may require a prolonged course of stent placement. Sealing of persistent pancreatic fistula with cyanoacrylate glue has been described and is a part of the treatment protocol for managing pancreatic fistulae in certain centers [46].

#### 6.3.2. Nasocystic Drainage

Placement of a nasocystic drain following initial access into a peri-pancreatic fluid collection allows for interval access to the collection for interventions such as lavage and administration of antibiotics. This is of particular benefit in walled-off pancreatic necrosis, where multiple sessions are required to adequately clear necrotic tissue. Use of the nasocystic catheter to perform lavage with normal saline in between endoscopic sessions has been shown to reduce rates of super-infection in this setting [47].

## 7. Complications of Endotherapy

General complications include severe bleeding and perforation. Prevention of this requires adequate pre-intervention assessment of the presence of a mature wall, ensuring close proximity of the GI wall to the collection, and addressing any coagulation abnormalities.

Secondary infection prevention requires use of prophylactic antibiotics. We tend to use ciprofloxacin for a period of up to 7 days.

### 7.1. Pseudocysts

Endoscopic pseudocyst drainage is generally a safely performed procedure. With current techniques, experienced operators have perforation rates as low as 1.2%, with bleeding rates of less than 1%. Perforation appears to be more common with pseudocysts in the uncinate region of the pancreas. Migration of stents occurs in less than 1% of cases. Infection rates are in the region of 5% [48].

### 7.2. Pancreatic Necrosis

Endoscopic necrosectomy of walled-off pancreatic necrosis is a more involved undertaking and occurs in a patient population that is usually sicker than those with pseudocysts. As such, complication rates are higher, being as high as 26% in the largest studies. The most common complications are bleeding and perforation, occurring in 54% and 21%, respectively, in the GEPARD study, with a mortality rate of 7.5% [13]. Given that endoscopic necrosectomy by definition involves operation outside the confines of the GI tract, air embolism is a potential complication not seen with other peri-pancreatic fluid collections, with a rate of 8% [13]. This complication can potentially be overcome by use of CO_2_ instead of air for insufflation.

## 8. Recent Advances and Future Developments

### 8.1. Forward-Viewing EUS Biopsy Capability

The standard EUS imaging platform currently available on the market for therapeutic interventions is the curvilinear echoendoscope, which provides an oblique view of the imaged tissue region. This results in practical disadvantages in the management of peri-pancreatic fluid collections as the accessories are advanced out of the scope tip at an acute angle. The mechanical force of the accessory onto the structure to be targeted tends to push the scope away from the area of interest, creating problems with adequate visualization and maintenance of direction. In addition, the angle of the scope tip that needs to be maintained for appropriate positioning is often problematic from an anatomical perspective. A forward-viewing echoendoscope with a working channel in line with the scope shaft has been developed to overcome these problems (Olympus). Although not yet commercially available, the limited data available on its usefulness in a variety of interventional procedures is encouraging [49]. It has proven effective in allowing drainage of pancreatic pseudocysts when use of the oblique-viewing echoendoscope has failed for technical reasons [50].

### 8.2. Use of SEMS

One drawback of using plastic stents to maintain the tract between a fluid collection and the enteric lumen is the tendency of these stents to migrate or become blocked. Self-expanding metal stents have been used in an attempt to overcome these problems [51], although migration may still be an issue. New stent designs with a larger diameter (20–25 mm) have been developed for use in pancreatic necrosectomy, with the advantage of maintaining a tract through which an endoscope can pass over multiple sessions, without having to change the stent on each occasion [52].

## 9. Conclusion

Management of the complications of severe acute pancreatitis, and peri-pancreatic fluid collections in general, has come a long way over the last 20 years. The employment of endoscopic management for these conditions is becoming more widespread as technologies and techniques continue to evolve. The available evidence highlights the first-line role for endoscopic management is certain situations, such as pseudocyst drainage, with increasing support for its utility in treatment of conditions such as infected pancreatic necrosis, in the appropriate clinical setting. The role of endoscopic management will continue to be refined as more long-term data becomes available, and management algorithms are more solidly established. The need to better define evidence-based optimal practice, and to develop appropriate device technology, provides fertile ground for clinical researchers and equipment manufacturers alike in advancing this cutting-edge area of endoscopy.

## Figures and Tables

**Table 1:** Atlanta Classification of SAP—pathomorphological entities.

Entity	Features

Pancreatic necrosis	Diffuse or focal areas of non-viable pancreatic parenchyma

Pancreatic abscess	Circumscribed intra-abdominal collection of pus containing little or no pancreatic necrosis

Acute fluid collection	Fluid collections located in or near the pancreas, lacking a surrounding wall of granulation/fibrous tissue

Acute pseudocyst	Collection of pancreatic juice surrounded by wall of granulation/fibrous tissue
